# Extracellular vesicles and melanoma: New perspectives on tumor microenvironment and metastasis

**DOI:** 10.3389/fcell.2022.1061982

**Published:** 2023-01-10

**Authors:** Alberto Benito-Martín, Miriam Galvonas Jasiulionis, Susana García-Silva

**Affiliations:** ^1^ Facultad de Medicina, Unidad de Investigación Biomédica, Universidad Alfonso X El Sabio (UAX), Villanueva de la Cañada, Spain; ^2^ Departamento de Farmacologia, Escola Paulista de Medicina, Universidade Federal de São Paulo, São Paulo, Brazil; ^3^ Microenvironment and Metastasis Laboratory, Molecular Oncology Programme, Spanish National Cancer Research Center (CNIO), Madrid, Spain

**Keywords:** extracellular vesicles, melanoma, vesicular cargo, microenvironment, pre-metastatic niche, metastasis, melanosomes

## Abstract

Secreted extracellular vesicles (EVs) are lipid bilayer particles without functional nucleus naturally released from cells which constitute an intercellular communication system. There is a broad spectrum of vesicles shed by cells based on their physical properties such as size (small EVs and large EVs), biogenesis, cargo and functions, which provide an increasingly heterogenous landscape. In addition, they are involved in multiple physiological and pathological processes. In cancer, EV release is opted by tumor cells as a beneficial process for tumor progression. Cutaneous melanoma is a cancer that originates from the melanocyte lineage and shows a favorable prognosis at early stages. However, when melanoma cells acquire invasive capacity, it constitutes the most aggressive and deadly skin cancer. In this context, extracellular vesicles have been shown their relevance in facilitating melanoma progression through the modulation of the microenvironment and metastatic spreading. In agreement with the melanosome secretory capacity of melanocytes, melanoma cells display an enhanced EV shedding activity that has contributed to the utility of melanoma models for unravelling EV cargo and functions within a cancer scenario. In this review, we provide an in-depth overview of the characteristics of melanoma-derived EVs and their role in melanoma progression highlighting key advances and remaining open questions in the field.

## Brief introduction on EVs and melanoma

Extracellular vesicles (EVs) are produced by almost all cell types and organisms studied. Although first evidences of vesicle release were established around 1950 (Yates et al., 2022), the detailed studies identifying secreted products from reticulocytes around 1980 (Sullivan et al., 1976; Pan and Johnstone, 1983) triggered a thriving and exciting research field with multiple diversifications in the following decades. The International Society of Extracellular Vesicles (ISEV) defines EVs as lipid bilayer particles without functional nucleus and naturally released from cells (Théry et al., 2018). These vesicles resemble their cell of origin in terms of their protein, lipid, and nucleic acid content (van Niel et al., 2018). EV physiological functions include antigen presentation, regulation of programmed cell death, angiogenesis, inflammation, coagulation, and fetus-mother communication during pregnancy (Yáñez-Mó et al., 2015; Yates et al., 2022). Nucleic acids carried by EVs including DNA, mRNA, microRNA (miRNA) and other RNA species are currently under extensive characterization due to their implication in pathogenesis and their potential as disease biomarkers (O'Brien et al., 2020). There are compelling evidences pointing to alterations in the recipient cells due to protein and RNA transfer, but many aspects of extracellular vesicle biology remain to be elucidated, including their actual DNA or RNA packaging (O'Brien et al., 2020). EVs capacity to exchange components between cells and to act as signaling vehicles in cellular and tissue homeostasis, but also in pathological scenarios, has driven most of EV-research efforts. Intercellular communication is a key feature of cancer progression (Minciacchi et al., 2015a) and metastasis (Wortzel et al., 2019). Furthermore, EVs role in the tumor microenvironment is crucial (Hou and Chen, 2021). In this review, we focus on melanoma, a type of skin cancer that develops from melanocytes following the accumulation of driven mutations (Schadendorf et al., 2018). According to the American Cancer Society, melanoma accounts for only about 1% of skin cancers but causes a large majority of skin cancer-related deaths. A large number of studies indicate that malignant melanoma risk correlates with individual characteristics and exposure to ultraviolet light (Shain and Bastian, 2016). Metastatic disease accounts for 90% of cancer-related deaths in those patients, although this percentage is expected to drop due to the implementation of immune checkpoint inhibitors-based treatments. Considering the role of EVs in the formation of pre-metastatic niches (PMNs) (Peinado et al., 2017) and in metastatic disease (Becker et al., 2016), we require a deeper understanding of the melanoma-EVs interplay in these processes. In 1988, Taylor et al. described how melanoma cells release intact portions of their plasma membranes in the form of vesicles, and how highly metastatic cells released vesicles more efficiently compared with poorly metastatic cells (Taylor et al., 1988). Subsequently, the role of melanoma-derived vesicles was described as crucial for melanoma invasion *in vitro* (Schnaeker et al., 2004). Moreover, a seminal study in the field demonstrated that exosomes were key determinants for the PMN cascade of events *in vivo* (Peinado et al., 2012). In addition, melanoma-derived EVs significantly and extensively influence a broad range of immune cells. In the following sections, we will extensively cover the heterogeneity, cargo and role of melanoma-derived vesicles in normal cell homeostasis and in cancer providing an in-depth analysis of the crucial aspects participated by EVs in melanoma progression.

## Melanoma-derived EV heterogeneity

Vesicle release occurs in all studied cells. However, the heterogeneity in size, composition and subcellular origin of those vesicles hampers a systematic understanding in the field. The ISEV leads an effort to homogenize and standardize EV nomenclature, in order to clarify different terminologies applied during the past decades in EVs studies. In 2018, Minimal Information for Studies of Extracellular Vesicles (MISEV) guidelines established a consensus position in the EV field, now followed by most of the community and editorial boards (Théry et al., 2018). This consensus position defined EV as particles without a functional nucleus naturally released from cells, delimited by a lipid bilayer and unable to replicate. MISEV2018 suggested categorizing EVs based on their physical condition (e.g., small EVs *versus* middle/large EVs), their biochemical composition (e.g., CD63^+^/CD81^+^ EVs), their physiological condition (e.g., hypoxic EVs) or their cell of origin. However, terms like exosomes, oncosomes or microvesicles still retain certain utility, as they tried, in their uniqueness, to reflect the rich heterogeneity included in the term extracellular vesicles.

### Small extracellular vesicles

Small EVs (sEVs) size range is 30–200 nm, including the vesicles named and characterized typically as “exosomes”. Exosomes are intraluminal vesicles formed by the inward budding of endosomal membrane during the maturation of multivesicular bodies (MVBs) and secreted upon fusion of MVBs with the plasma membrane. Their biogenesis, characteristics and release have been extensively described (Colombo et al., 2014; Lo Cicero et al., 2015a; Yáñez-Mó et al., 2015). Most of the research around melanoma and EVs is focused on this sEV type. It has been largely reviewed since 2001, when Amigorena discussed the usage of human dendritic cell (DC)-derived exosomes for cancer immunotherapy (Amigorena, 2001). Apart from their promising contribution to new immunotherapy strategies, small EVs/exosomes and melanoma have been studied in multiple contexts: disease biomarkers (Alegre et al., 2015), metastasis (Weidle et al., 2017), obesity-cancer link (Clement et al., 2017), drug resistance (Namee and O'Driscoll, 2018), cancer-associated fibroblasts (Shelton et al., 2021) and many others.

### Large extracellular vesicles

Large extracellular vesicles (lEVs) define lipidic layer particles with a size range above 200 nm, including vesicles traditionally labeled as microvesicles, ectosomes, microparticles, oncosomes, migrasomes, and other denominations. In a seminal article, Peter Wolf described lEVs as subcellular material originating from platelets and named them as “platelet dust” (Wolf, 1967). In the following years, ectocytosis, a process allowing the release of plasma membrane vesicles, in immune cells was described (Stein and Luzio, 1991). lEVs are generated by the outward budding and fission of the plasma membrane and the subsequent release into the extracellular milieu (van Niel et al., 2018). lEVs have a role in cell–cell communication and when this exchange happens in the cancer context, they have been generally denominated oncosomes. In the seminal study by Al-Nedawi and colleagues, oncosomes were identified as the vehicle for oncogenic epidermal growth factor receptor vIII (EGFRvIII) transfer between glioma cells, spreading the transformed phenotype (Al-Nedawi et al., 2008). In melanoma, Fas ligand (FasL)-bearing microvesicles trigger Fas-dependent apoptosis of lymphoid cells (Andreola et al., 2002). Tumor necrosis factor superfamily member 14 (TNFSF14)/LIGHT^+^ lEVs regulate T-cell responses to tumor cells (Mortarini et al., 2005) and exhibit a role in immune suppression (Valenti et al., 2007).

There are at least three subtypes of large extracellular vesicles that demand attention in the cancer setting:

*Migrasomes,* with a diameter range between 500 and 3000 nm, display an oval shape, contain smaller vesicles and have a role in tumor cell migration. In 2015, Ma et al. found that B16 mouse melanoma migrating cells left behind a ring-like organelle derived from retraction fibers (Di Daniele et al., 2022). Upon exposure to mild mitochondrial stresses, damaged mitochondria are transported into migrasomes and subsequently discarded from migrating cells in a polarized way. This newly described mechanism, mytocytosis, plays an important role in maintaining mitochondrial performance and links mitochondrial homeostasis with cell migration (Jiao et al., 2021).

*Apoptotic Bodies* (1–5 μm) are released by cells after a triggered collapse that results in nuclear fragmentation, increased membrane permeability and externalization of phosphatidylserine (PS). Apoptosis plays a key role in development and homeostasis (Li et al., 2022a). During this highly orchestrated process of cell disintegration, proteins, lipids and nucleic acids fragments are distributed in vesicles (Crescitelli et al., 2013).

*Large oncosomes* present a large size (1–10 μm) and carry abundant oncogenic cargo. They clearly differ in protein content from smaller EVs and show enrichment of enzymes involved in glucose, glutamine and amino acid metabolism (Minciacchi et al., 2015b). These lEVs alter the tumor microenvironment, promote disease progression (Di Vizio et al., 2009) and contribute to the spreading of oncogenic information, including the transfer of signal transduction complexes between tissues (Bertolini et al., 2019).

### The others

The concept “extracellular particles” (EPs) was proposed by ISEV “if confirmation of EV identity cannot be achieved according to the minimal requirements” (Théry et al., 2018). There are certain biological entities released by cells that do not comply certain aspects of EVs biology. Among these EPs, exomeres have been described and studied in melanoma (Zhang et al., 2018). Exomeres are non-membranous nanoparticles of up to 50 nm in size. They differ in protein, RNA, DNA and lipid content compared to small and large EVs (Anand et al., 2021). Exomere proteomic profiling revealed an enrichment in metabolic enzymes and hypoxia, microtubule and coagulation-related proteins as well as specific pathways, such as glycolysis and mTOR signaling. They can transfer functional cargo, although their origin has not been explored yet (Zhang et al., 2019). Recently, another particle type below 50 nm in size has been defined, the supermere (Zhang et al., 2019; Zhang et al., 2021). The identification of these novel nanovesicles proves how far we are from fully assessing the complexity and heterogeneity of the extracellular released content.

## Melanoma EV cargo

**FIGURE 1:**
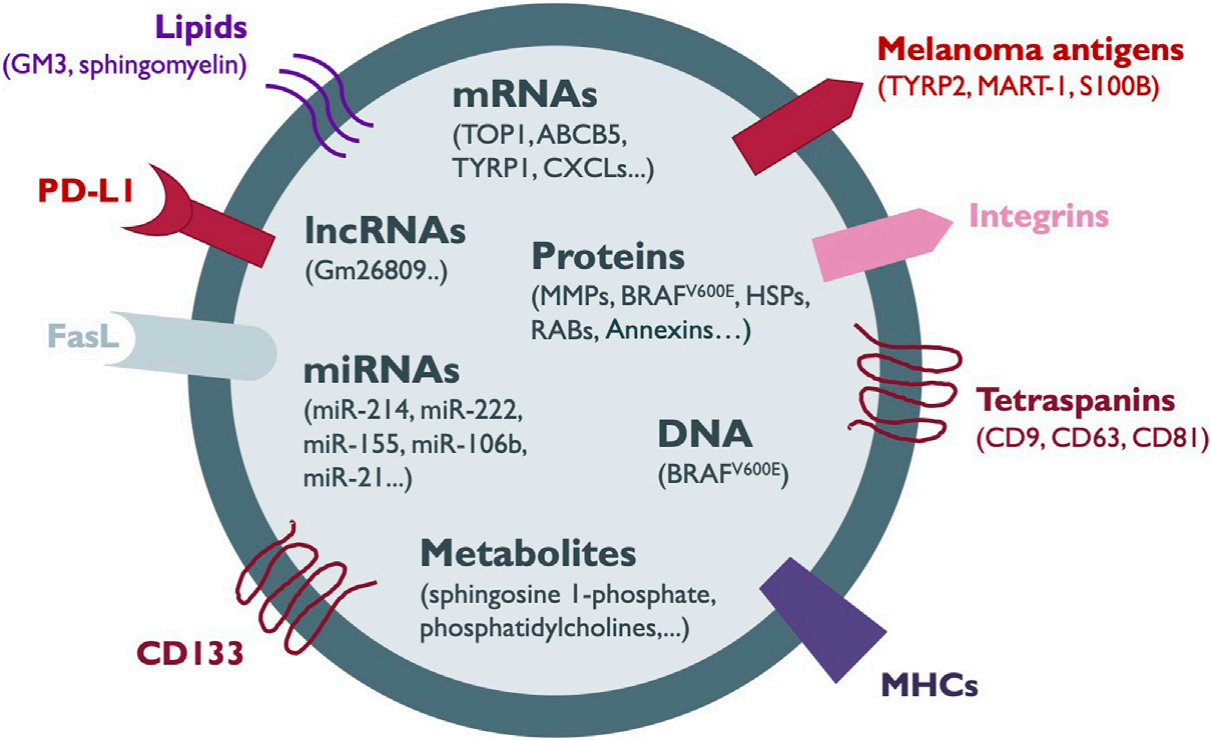
Melanoma-secreted EV cargo. The components of the heterogeneous cargo of melanoma EVs comprise membrane proteins such as tetraspanins, integrins, melanoma antigens, CD133, Fas ligand (FasL), immune checkpoint proteins such as PD-L1 and antigen-presenting complexes such as MHC-I. In addition, EVs can contain other proteins including metaloproteinases (MMPs), oncogenes, RAB proteins, heat shock proteins (HSPs) and annexins. Several types of nucleic acid species such as DNA, messenger RNA (mRNA), microRNAs (miRNAs) and long non-coding RNAs (lncRNAs) together with lipids, and metabolites are also shuttled in melanoma EVs. Most of the information about the EV cargo has been obtained from the analysis of sEVs.

Extracellular vesicles (EVs) secreted by normal and tumor cells contain a variety of bioactive molecules, such as RNA (messenger RNAs, miRNAs, long non-coding RNAs), DNA (single-stranded, double-stranded and mitochondrial), proteins, lipids and metabolites (Figure 1; Table 1). EVs containing such molecular information can be delivered over long distances to recipient cells or tissues within the body (Pegtel and Gould, 2019; Kalluri and LeBleu, 2020). Furthermore, the transfer of whole organelles in EVs, such as mitochondria, has also been reported (Hayakawa et al., 2016). The function of recipient cells is modulated by the transferred cargo, and the effects are dependent on the type and molecular composition of EV content, which in turn is determined by the cell of origin (Raposo and Stoorvogel, 2013). In cancer, tumor-derived EVs influence several key processes for tumor progression, such as the establishment of PMNs, angiogenesis, cell migration and invasion, and suppression of immune responses (Kanada et al., 2016; Hoshino et al., 2015; Sheehan and D'Souza-Schorey, 2019).

**TABLE 1:** Cargo of melanoma-derived EVs. EVs released by melanoma cells display a heterogeneous cargo composed by proteins, mRNA, miRNA, non-coding RNAs, DNA, lipids and metabolites. Some of these molecules are commonly found in vesicles from multiple origins but others are so far specific of melanoma cell types.

Cargo	Findings
**mRNAs**	
*TOP1*	Enriched in EVs derived from human melanoma cells (A375 and SK-MEL-28) compared to primary melanocytes Xiao et al. (2012)
*ABCB5, TYRP1*	Reduced in EVs derived from human melanoma cells (A375 and SK-MEL-28) compared to primary melanocytes Xiao et al. (2012)
*CXCL1, CXCL2, CXCL8*	Enriched in EVs from human melanoma cells (A-375, SKMEL-28, and C-32) compared to primary melanocytes Bardi et al. (2019)
*PD-L1*	Reduced in plasma EVs in patients responding to anti-PD-1 treatment, and increased levels in subjects with disease progression Del Re et al. (2018)
**Proteins**	
p120 catenin, Nedd5, PGRL, ezrin, radixin	Identified in exosomes derived from human melanoma cell lines (SK-MEL-28 and MeWo) Mears et al. (2004)
Annexin A1, annexin A2, syntenin-1, HAPLN1	Enriched in EVs from the human metastatic melanoma cells A375 compared to primary melanocytes Xiao et al. (2012)
CD44, MAPK4K, GTP-binding proteins, ADAM10	Enriched in the human metastatic melanoma cell line FEMX-I Rappa et al. (2013)
FAK1, SRC, Rac1, RhoA, Cdc42, EGFR, EPHB2	Enriched in exosomes derived from human metastatic melanoma cells (A375M and 1205Lu) compared to non-tumorigenic (MNT-1, G-1 and 501mel) and tumorigenic but non-metastatic cells (Daju and SKMel-28) Lazar et al. (2015)
TYRP2, VLA-4, HSP70	Enriched in exosomes from plasma of melanoma patients with advanced disease compared to healthy subjects Peinado et al. (2012)
MIA, S100B	Enriched in exosomes isolated from the sera of stage IV melanoma patients compared to healthy controls Alegre et al. (2016)
CD14, PON1, PON3, APOA5	Different content in EVs from plasma of melanoma patients at different stages (0-IV) Paolino et al. (2021)
PD-L1	Its level on EVs correlates with therapeutic response to immune checkpoint inhibitors in melanoma Cordonnier et al. (2020)
PD-1	Its enrichment on melanoma EVs was suggested as a possible resistance mechanism Chen et al. (2018); Poggio et al. (2019); Serratì et al. (2022)
MCSP, MCAM, LNGFR, ErbB3	EV level changes after targeted therapies in melanoma patients Wang et al. (2020a)
**miRNAs**	
let-7c, miR-138, miR-125b, miR130a, miR-34a, miR- 196a, miR-199b-3p, miR- 25, miR-27a, miR-200b, miR-23b, miR-146a, miR- 613, miR-205, miR-149, and others	Enriched in human melanoma cells (A375) compared to melanocytes Xiao et al. (2012)
miR-214-3p, miR-199a-3p, miR-155-5p	Enriched in exosomes of human melanoma cell lines (A375, MML-1 and SKMEL-28) Lunavat et al. (2015)
miR-100-5p, miR-99b-5p, miR-221-3p, miR-24-3p, miR-125b-5p, and others	Enriched in human melanoma cells (WM9, WM35 and WM902B) compared to melanocytes Gerloff et al. (2020)
miR-222	Delivery of miR-222 by EVs from metastatic melanoma cells increases the tumorigenicity of less aggressive melanoma cells Felicetti et al. (2016)
miR-106b-5p	The transfer of this miRNA by exosomes to melanocytes promotes *in vitro* cell migration, invasion, and *in vivo* lung metastasis Luan et al. (2021)
miR-21	Exosomal miR-21 from murine B16 melanoma cells delivered to stromal fibroblasts increases MMP expression, favoring melanoma progression Wang et al. (2020b)
miR-92b-3p, miR-182-5p, miR-183-5p	Enriched in melanoma *versus* melanocyte derived EVs and in the plasma of melanoma patients compared to healthy donors Gerloff et al. (2020)
miR-17, miR-19a, miR-21, miR-126, miR-149	Enriched in plasma EVs of patients with metastatic sporadic melanoma compared with unaffected control subjects Pfeffer et al. (2015)
miR-125b	Reduced levels in exosomes obtained from serum of patients with advanced melanoma Tengda et al. (2018)
miR-532-5p, miR-106	Enriched in exosomes from serum of melanoma patients compared to healthy individuals Léveillé and Baglio, (2019)
miR-191, let-7a	Enriched in EVs from stage-I melanoma patients compared to healthy individuals Xiao et al. (2016)
**lncRNAs**	
Gm26809	The transfer of this exosomal lncRNA from melanoma cells to fibroblasts reprograms fibroblasts into cancer-associated fibroblasts Hu and Hu, (2019)
**DNA**	
BRAF^V600E^	Exosomal mutant BRAF as a promising biomarker for monitoring BRAFi therapy Zocco et al. (2020); Detection in lymphatic drainage derived EVs from stage III melanoma patients correlates with risk of relapse García-Silva et al. (2019)
**Lipids**	More saturated and shorter fatty acid chains in EVs from low metastatic cells (LCP) compared to high metastatic (SKMel28) melanoma cells Lobasso et al. (2021)
**Metabolites**	Decreased levels of palmitoyl carnitine, sphingosine 1- phosphate, elaidic carnitine, phosphatidylcholines, phosphatidylethanolamines, and glycosphingolipid ganglioside GM3 in exosomes from serum of melanoma patients compared to that of healthy individuals Palacios-Ferrer et al. (2021)

The diversity of EV molecular content is also determined by their biogenesis. In sEVs such as exosomes, Endosomal Sorting Complex Required for Transport (ESCRT) proteins, ESCRT-associated proteins (such as ALIX) and RNA-binding proteins are involved in the specific incorporation of proteins and RNAs (Henne et al., 2011; Bissig and Gruenberg, 2014; Villarroya-Beltri et al., 2014). Tetraspanins such as CD9, CD63, CD81 and CD82 are enriched in exosomes, and also participate in specific protein loading (Théry et al., 2002). In microvesicles (MVs), GTPases seem to have an important role both in its formation and cargo recruitment (Tricarico et al., 2017). MVs are shed from plasma membrane regions enriched in integrins, Human Leucocyte antigen (HLA) molecules and proteolytic enzymes. These molecules are among common MV cargo (Vittorelli, 2003; Muralidharan-Chari et al., 2009). MVs also contain unique glycan proteins, as fucosylated glycoproteins and complex *N*-glycans with bisecting GlcNAc (Surman et al., 2018). The content of apoptotic bodies includes condensed nuclear chromatin and cytoplasmic components (Kerr et al., 1972). In tumors, it was observed that apoptotic bodies can transfer DNA to neighboring cells (Bergsmedh et al., 2001; Yan et al., 2006).

### mRNA cargo of melanoma-derived EVs

By analyzing the mRNA content in exosomes from melanocytes and melanoma cell lines, Xiao and colleagues found melanoma-expressed mRNA transcripts in melanoma-derived EVs (Xiao et al., 2012). Thousands of exosomal mRNAs, such as DNA topoisomerase I (TOP1), ATP-binding cassette, sub-family B, member 5 (ABCB5) and tyrosinase-related protein 1 (TYRP1), were identified as differentially enriched in sEVs from melanoma cells compared to melanocytes and correspond to genes associated with melanoma progression and metastasis (Ryan et al., 2010; Journe et al., 2011; Soengas and Hernando, 2017). Increased levels of inflammation-related mRNAs, such as C-X-C motif chemokine ligand 1, 2 and 8 (CXCL1, CXCL2 and CXCL8) mRNAs, were also identified in melanoma-derived EVs compared to those derived from primary melanocytes, which may be related to a pro-inflammatory role (Bardi et al., 2019).

Using clinical samples, decreased levels of PD-L1 mRNA were found in plasma-derived EVs in patients responding to immune checkpoint inhibitors (ICI) treatment, whereas increased levels were observed in subjects with disease progression (Del Re et al., 2018). Additionally, for a comprehensive assessment of EV-RNAs, Shi and colleagues performed a transcriptome analysis of plasma-circulating EVs in melanoma patients after ICI treatment. Transcripts enriched in EVs were related to ICI resistance, melanoma progression, and response to ICI therapy (Shi et al., 2020). These studies point to the use of EVs-associated mRNAs as predictive markers of ICI responsiveness.

### Melanoma-secreted EV proteins

The protein content of melanoma-derived EVs, essentially exosomes, has been profiled from several cell lines and plasma samples (Surman et al., 2019a). Interestingly, the available data on melanoma-derived EVs have revealed the presence of several oncogenic proteins (Mears et al., 2004; Xiao et al., 2012; Lazar et al., 2015). Although proteins carried on melanoma-derived EVs have emerged as promising melanoma biomarkers, the amount of clinical data demonstrating their utility is still very limited.

The study from Mears and colleagues was the first to demonstrate the presence of proteins in melanoma-derived EVs (Mears et al., 2004). The protein analysis was performed in exosomes isolated from cell supernatants and cell lysates of two melanoma cell lines (MeWo and SK-MEL-28). These results not only confirmed the existence of MHC-I and the tumor-associated antigens MART-1 and MUC-18 in melanoma-derived EVs, but they revealed the presence of several proteins, such as p120 catenin, Nedd5, prostaglandin regulatory-like protein (PGRL), ezrin and radixin. Around 50% of proteins were found both in cell lysates and in the corresponding exosome fraction. They also observed a general reduction of lysosomal and mitochondrial proteins in exosomes compared to cell lysates.

In 2012, Xiao and collaborators identified 11 proteins differentially enriched in exosomes from the metastatic melanoma cell line A375 compared to normal melanocytes HEMa-LP (Xiao et al., 2012). Annexin A1, annexin A2, syntenin-1 and hyaluronan and proteoglycan link protein 1 (HAPLN1) were among these proteins, all involved in processes related to melanoma progression, such as angiogenesis, cell invasion, migration and metastasis. Another study also identified annexin A2 in prominin-1/CD133-enriched sEVs. Other proteins found in these sEVs include the pro-metastatic proteins CD44, MAPK4K, GTP-binding proteins, and ADAM10 (Rappa et al., 2013).

Interestingly, EV protein content depends on the melanoma type and progression stage since only 25% of the sEV-associated proteins were shared between 7 cell lines with different tumorigenic and metastatic potential (Lazar et al., 2015). Gene ontology analysis showed that proteins found in EVs from metastatic melanoma cells were enriched in biological functions related to tumor aggressiveness, such as cell migration, regulation of apoptosis and angiogenesis. In addition, exosomes derived from metastatic cell lines were able to increase the migration of less aggressive cell lines. They also found several immunosuppressive proteins, such as galectins (LGALS1 and LGALS3) and 5′-nucleotidase (NT5E) in melanoma-derived exosomes, indicating their possible role in tumor escape from immune surveillance. In the same direction, the whole proteome analysis of exosomes derived from the B16 melanoma mouse model revealed proteins related to molecular processes including cellular movement, proliferation, and cell morphology (Gyukity-Sebestyén et al., 2019). More recently, Guerreiro and colleagues described that sEVs from different types of human cancer (oral squamous cell carcinoma, pancreatic ductal adenocarcinoma, and melanoma brain metastasis) share 25% of protein content, mostly linked to tumor processes (Guerreiro et al., 2020).

A variety of integrins and other adhesion molecules are also present in melanoma-secreted sEVs, similarly to other tumor-derived EVs (Hoshino et al., 2015; Paolillo and Schinelli, 2017; García-Silva et al., 2021). They appear to be important for the targeting of recipient tissues and cells.

Small and large melanoma-derived EVs carry tissue factor (TF) and other clotting associated proteins that are absent in non-transformed melanocytes (Lima et al., 2011; García-Silva et al., 2019), suggesting that promoting coagulation is a differential feature of melanoma-derived vesicles.

As exemplified above, in most studies, the main source of EVs was conditioned culture media from melanoma cell lines. Studies evaluating exosomes isolated from the blood of melanoma patients are still limited. In 2012, Peinado and collaborators compared exosomes isolated from plasma of melanoma patients with those from healthy donors and showed that the total amount of protein per particle in plasma-circulating sEVs significantly increases through melanoma progression (Peinado et al., 2012). Furthermore, a significant enrichment of TYRP2, VLA-4, and HSP70 was found in advanced stages of the disease compared to healthy subjects. In another study, the levels of Melanoma inhibitory activity protein (MIA), S100B and tyrosinase-related protein 2 (TYRP2), known melanoma biomarkers, were evaluated in exosomes isolated from the sera of stage IV melanoma patients and healthy controls. The enrichment of MIA and S100B in exosomes of melanoma patients indicated their potential value as diagnostic and prognostic markers (Alegre et al., 2016). Different content of some proteins, such as CD14, PON1, PON3 and APOA5, was revealed in small EVs from plasma of melanoma patients at distinct stages (0-IV), suggesting their potential use as biomarkers to monitor the disease (Paolino et al., 2021).

Regarding protein cargo, few information is available in uveal melanoma-derived EVs. Similar to cutaneous melanoma-derived EVs, proteins associated to cell proliferation, apoptosis, invasion and cancer cell metabolisms such as annexins, galectins, dehydrogenases, chaperones and integrins have been identified in uveal melanoma-secreted ectosomes (Surman et al., 2019b).

The presence of PDL-1 and FasL has been reported in tumor-derived EVs as a mechanism to inhibit the immune response against the tumor (Andreola et al., 2002; Chen et al., 2018). In melanoma, the level of EV-associated PD-L1 does not correlate with clinical pathological characteristics (Cordonnier et al., 2020). However, it was correlated with therapeutic response to immune checkpoint inhibitors and EVs enriched with PD1 were suggested as a possible resistance mechanism (Chen et al., 2018; Poggio et al., 2019; Serratì et al., 2022). Another study also showed changes in specific proteins, such as melanoma chondroitin sulphate proteoglycan (MCSP), melanoma cell adhesion molecule (MCAM), low-affinity nerve growth factor receptor (LNGFR) and receptor tyrosine-protein kinase (ErbB3), as relevant EV cargo during and after targeted therapies (Wang et al., 2020a).

### MiRNAs in melanoma EVs

Since Valadi and collaborators showed that miRNAs carried by EVs could be transferred to target cells resulting in gene expression alterations, its role in cell communication gained more attention (Valadi et al., 2007). In melanoma, most available data regarding miRNA as EV cargo came from cell lines and few studies have addressed miRNA cargo in circulating EVs in melanoma patients.

Comparing A375 cell line and HEMa-LP melanocytes, Xiao and colleagues identified 130 miRNAs enriched and 98 miRNAs decreased in melanoma-derived EVs, being a significant fraction associated with cancer (Xiao et al., 2012). Interestingly, miRNAs enriched in melanoma EVs such as let-7c, miR-138, miR-125b, miR130a, miR-34a, miR-196a, miR-199b-3p, miR-25, miR-27a, miR-200b, miR-23b, miR-146a, miR-613, miR-205, miR-149, have already been associated with melanoma metastasis. A comparative analysis of the RNA cargo between different EV subtypes using A375, MML-1 and SK-MEL-28-derived EVs, revealed the enrichment of certain miRNAs in exosomes, including miR-214-3p, miR-199a-3p and miR-155-5p, all associated with melanoma progression and highly expressed in melanoma tumors compared to benign nevi (Lunavat et al., 2015). In another study comparing exosome content from human melanoma cell lines (WM9, WM35 and WM902B) and melanocytes (NHEM), 34 miRNAs were found differentially enriched, such as miR-100-5p, miR-99b-5p, miR-221-3p, miR-24-3p, and miR-125b-5p (Gerloff et al., 2020). Although few reports share miRNA findings in melanoma EVs (Pfeffer et al., 2015; Wozniak et al., 2017; Zhou et al., 2018a; Li et al., 2019), several studies support the role of EV-loaded miRNAs in melanoma cell invasion, migration, proliferation and tumor progression (Müller and Bosserhoff, 2008; Nyholm et al., 2014; Felicetti et al., 2016; Xiao et al., 2016; Li et al., 2019). Moreover, melanoma-derived EVs were shown to modify the behavior of recipient cells by the transfer of miRNAs. Delivery of miR-222 by EVs from metastatic melanoma cells to less aggressive melanoma cells increase their tumorigenicity (Felicetti et al., 2016). miR-106b-5p, enriched in exosomes from serum of melanoma patients, is transferred to melanocytes, inducing epithelial-to-mesenchymal transition, promoting *in vitro* cell migration, invasion, and *in vivo* lung metastasis (Luan et al., 2021). Using the murine B16 melanoma cell line, Wang and colleagues showed that the transfer of exosomal miR-21 to stromal fibroblasts led to TIMP3 inhibition, resulting in increased expression of matrix metalloproteases (MMPs) which favored tumor progression (Wang et al., 2020b).

Remarkably, miRNAs such as miR-92b-3p, miR-182-5p and miR-183-5p were found differentially loaded in melanoma *versus* melanocyte-derived sEVs and were also enriched in the plasma of melanoma patients compared to healthy donors (Gerloff et al., 2020).

In a study comparing the miRNA content in plasma-derived exosomes from patients with metastatic melanoma, familial melanoma or healthy subjects, Pfeffer and collaborators reported a number of differentially enriched miRNAs, including miR-17, miR-19a, miR-21, miR-126, and miR-149 (Pfeffer et al., 2015). Lower levels of miR-125b in exosomes obtained from serum of patients were shown to be associated with advanced melanoma disease (Alegre et al., 2014). Higher levels of miR-532-5p and miR-106 were found in exosomes from serum of melanoma patients compared to healthy individuals. The combination of these two miRNAs showed a higher efficiency to diagnose melanoma patients than the known biomarkers Lactate dehydrogenase (LDH), MIA, or S100B alone or in combination (Tengda et al., 2018). Among other miRNAs found up-regulated in serum sEVs from melanoma patients are miR-191 and let-7a, which were shown to distinguish stage-I melanoma patients from healthy individuals (Xiao et al., 2016).

Other non coding RNAs, such as long non-coding RNAs (lncRNAs), can also be loaded into EVs and may play a role in reprogramming the tumor microenvironment (Qu et al., 2016; Léveillé and Baglio, 2019). For example, the lncRNA Gm26809 shuttled in melanoma exosomes was involved in the reprogramming of fibroblasts into cancer-associated fibroblasts (CAFs) (Hu and Hu, 2019).

### Melanoma EV-associated DNA

Many studies have analyzed circulating tumor DNA (ctDNA) in plasma of melanoma patients, but not in EV-associated DNAs. By determining the BRAF gene status on EVs from plasma samples of metastatic melanoma patients at the beginning and during therapy with BRAF inhibitors, Zocco and collaborators showed that EV-associated DNA can be a better alternative to ctDNA for detection of mutant BRAF in these patients (Zocco et al., 2020). Wild type BRAF and BRAF V600E mutation have also been detected in lymphatic drainage-derived EVs (García-Silva et al., 2019). On the other hand, mitochondrial DNA (mtDNA) content in melanoma-derived vesicles have not been addressed in detail although it is expected to be represented (Cheng et al., 2020).

### Lipids in melanoma EVs

The analysis of lipid content in EVs derived from melanoma cell lines with low and high metastatic potential (LCP and SK-MEL28, respectively) showed more saturated and shorter fatty acid chains in low metastatic cells compared to high metastatic cells (Lobasso et al., 2021). Of note, the lipid composition of cell and exosome membranes is altered by the microenvironmental pH (Parolini et al., 2009).

### Metabolites in melanoma EVs

The metabolomic profile of exosomes from serum of melanoma patients compared to that of healthy individuals demonstrated decreased levels in several metabolites including palmitoylcarnitine, sphingosine 1-phosphate, elaidic carnitine, phosphatidylcholines, phosphatidylethanolamines and glycosphingolipid ganglioside GM3 (Palacios-Ferrer et al., 2021).

## EVs in normal homeostasis

There is plenty of evidence for the importance of extracellular vesicles in pathological states. From cancer interaction with the immune system (Pelissier Vatter et al., 2021) to cancer resistance to therapy (Palazzolo et al., 2022), from cardiovascular disease (de Abreu et al., 2020) to anaphylaxis (Fernández-Bravo et al., 2022), the EV role in pathogenesis and its potential use as biomarker have been well characterized, albeit there are still many questions to answer. However, EVs role in normal physiology or cellular and tissue homeostasis presents multiple pending questions. The first descriptions of EVs physiological roles rose around their contribution to immune system function and mostly were focused on antigen presentation (Raposo et al., 1996). In dendritic cells (DCs), protein cargo of sEVs derived from intestinal epithelial cells or other DCs are processed in the endocytic compartment similarly to antigens and then used in antigen presentation, thereby contributing to immune response regulation (Morelli et al., 2004; Mallegol et al., 2007).

Another interesting physiological process in which EVs seem to play a determinant role is embryogenesis. Small EVs participate in the secretion and processing of Wnt proteins, a family of morphogens with fundamental roles in homeostasis and cancer (Zhang and Wrana, 2014). Furthermore, lEVs generated and released by embryonic stem cells induce invasion of maternal tissue by the trophoblast, promoting embryo implantation (Desrochers et al., 2016), consolidating the relevance of EVs in development. Neural crest cells, unique to vertebrates, arise from the embryonic ectoderm germ layer, and in turn give rise to diverse cell lineages—including melanocytes, craniofacial cartilage and bone, smooth muscle, peripheral and enteric neurons and glial cells (Shakhova and Sommer, 2008). Recently, it has been described how migratory neural crest cells release and deposit CD63^+^ 30–100 nm particles and migrasomes into the extracellular environment and how inhibition of RAB27A docking in the membrane alters their migratory phenotype (Gustafson et al., 2022). These results indicate that sEVs release by neural crest cells is critical for neural embryonic migration.

## A role for EVs in normal melanocyte/skin physiology?

Melanocytes are located in the dermis and basal epidermis and display a clearly polarized and dendritic architecture. Melanocytes protect keratinocytes from UV radiation in the skin through the generation of a brown-black pigment, eu-melanin that is transferred from the melanocytes to the keratinocytes. Eu-melanin locates around the sun-exposed side of the nuclei in a cap-like fashion to shield the keratinocyte DNA from ultraviolet light (UV)-induced damage. The transfer of eu-melanin to keratinocytes occurs through melanosomes originated from endosomal membranes (Yardman-Frank and Fisher, 2021). Melanosomes are exclusive of animal cells, display a bilipidic membrane and their size is approximately 500 nm. They participate in the synthesis, storage, and transport of melanin (Wasmeier et al., 2008). Melanosome biogenesis occurs in four stages. Stage I consists of the formation of intraluminal vesicles and fibrils. In stage II, fibrils arrange in sheet-like structures and the vesicles adopt an ellipsoidal shape. During Stage III, melanin-synthesizing enzymes such as tyrosinase, TYRP1 and DCT/TYRP2 are transferred to the pre-melanosomes and melanin synthesis starts. During stage IV, the structure is covered by melanin, generating the mature melanosome (Delevoye et al., 2019). It is possible that melanosomes release could be polarized accordingly to the polarized cell organization of the melanocytes. Interestingly, it has been recently shown that Claudins, important proteins participating in polarity and secretory mechanisms, are involved in the transfer of melanosomes in a gold fish model (Liu et al., 2022). However, melanosome transfer *in vivo* is far from been understood in detail (Tadokoro and Takahashi, 2017; Li et al., 2020).

Remarkably, proteins traditionally associated with the biosynthesis, transport and release of EVs like RAB proteins (Fukuda, 2021), CD63, SNARE, and BLOC complexes (Ohbayashi and Fukuda, 1000) are involved in melanosome biogenesis but also in the biogenesis of other EVs. Remarkably, the RAB family of small GTPases promotes the progression of melanoma and other cancers (Li et al., 2018a). RAB1A, RAB5B, RAB7 and RAB27A, are highly expressed in melanoma cells. RAB27A depletion impairs exosome production, preventing bone marrow progenitors’ contribution to the formation of the PMN, and reducing tumor growth and metastasis as well (Peinado et al., 2012). RAB27A, together with melanophilin/SLAC2a, mediates the connection of melanosomes to actin filaments. Loss of RAB27A expression in melanoma cell lines inhibited their invasive phenotype (Guo et al., 2019).

Keratinocytes are the predominant cell type in the epidermis of the skin, the largest and most superficial organ of the body. It has been reported that keratinocytes release extracellular vesicles, mostly referred as exosomes, to the extracellular environment and use them to communicate with other skin cells and the immune compartment (Chavez-Muñoz et al., 2008; Kotzerke et al., 2013; Than et al., 2019). Recently, it has been appreciated how EVs secreted into interstitial spaces mediate the interchange between keratinocytes and melanocytes. Keratinocytes communicate with melanocytes *via* sEVs carrying miRNAs such as miR-203 and miR-3196 with the capacity to modulate pigmentation in addition to known molecules such as the α-melanocyte stimulating hormone (α-MSH) and the adrenocorticotropic hormone (ACTH) (Hushcha et al., 2021). These keratinocytes-derived vesicles increase tyrosinase activity, pigmentation genes and melanin content in recipient melanocytes to stimulate more melanosome production (Lo Cicero et al., 2015b). In another study, miRNA transference from keratinocytes to melanocytes was proposed as a mechanism for melanogenesis inhibition through MITF-H regulation (Kim et al., 2014).

**FIGURE 2:**
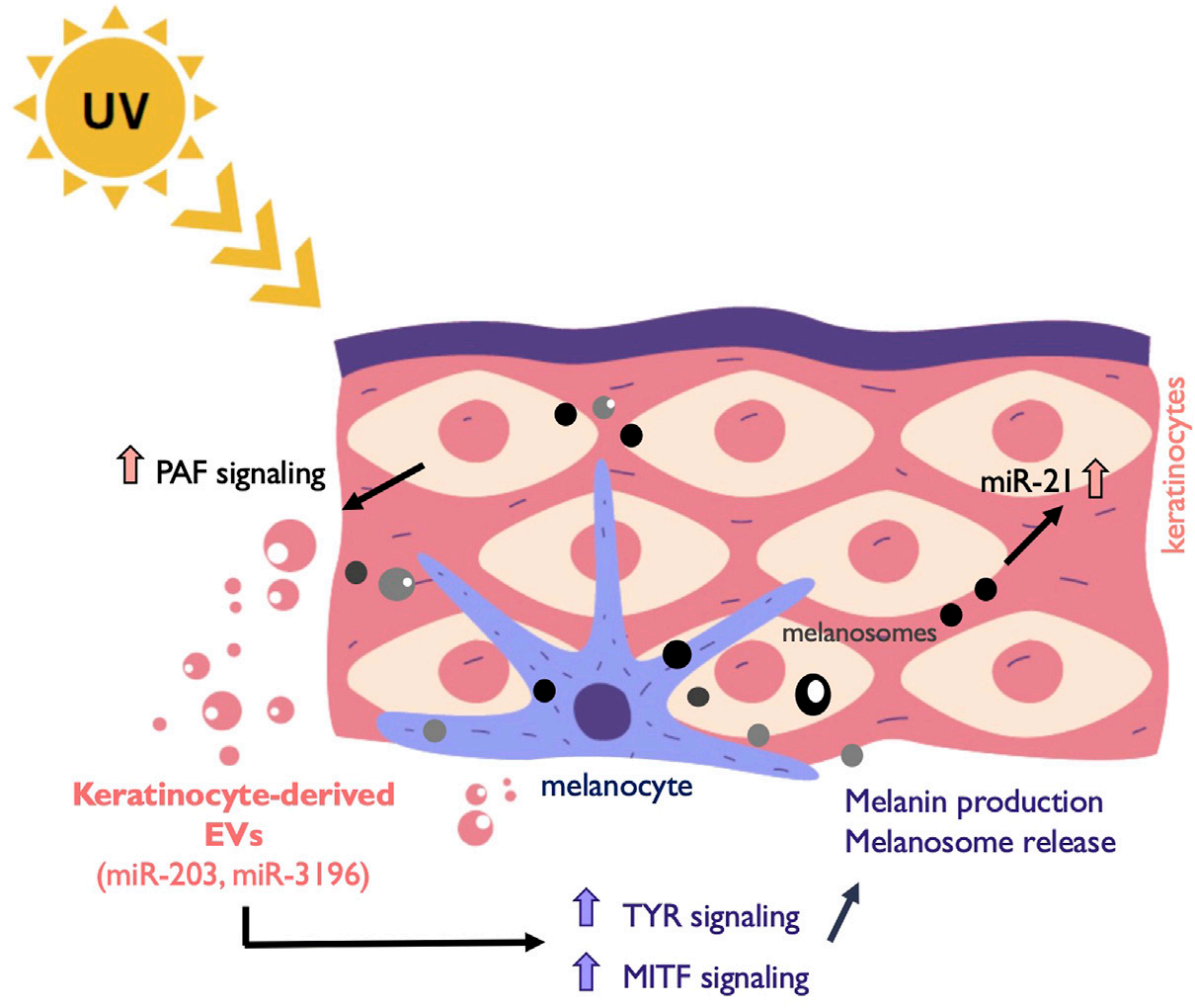
Melanocyte and keratinocytes communicate through EVs. Exposure to UV light induces among other responses the release of EVs from keratinocytes that are taken up by surrounding melanocytes. This EV release is induced by platelet-activating factor (PAF). Keratinocyte-derived cargo contains miRNAs such as miR-203 and miR-3196 that promote the upregulation of the melanogenesis master regulator MITF and other genes involved in the process such as Tyrosinase (TYR). Melanin is subsequently secreted in melanosomes to the epidermis and taken up by near-by keratinocytes. In addition, melanosome contains other cargo that induce anti-apoptotic signals in recipient cells through the upregulation of miR-21.

An important function of melanocytes-keratinocytes EV communication is related to the skin response to UV radiation, which causes skin pigmentation and, if not properly addressed, skin cancer. There are two basic types of ultraviolet rays that reach the earth’s surface—Ultraviolet A (UVA) and Ultraviolet B (UVB) rays. UVA light induces plasma membrane damage, which is repaired by lysosomal exocytosis followed by instant shedding of EVs from the plasma membrane (Wäster et al., 2020). The released EVs are incorporated by neighboring cells, leading to the activation of proliferation and anti-apoptotic signaling *via* miR-21. UVB radiation found in sunlight is essential for vitamin D production in humans. However, prolonged exposure can lead to a variety of pathologic effects including erythema, photoaging, inflammatory responses, and skin cancer (Narayanan et al., 2010). Keratinocytes receiving UVB release large EVs due to Platelet-Activating Factor (PAF) Signaling (Bihl et al., 2016). The interplay between keratinocytes and melanocytes mediated by extracellular vesicles under the influence of UV is not fully understood yet, but there are evidences suggesting a constitutive role for this exchange in cutaneous pigmentation regulation (Takano et al., 2020) (Figure 2).

In early melanoma, Dror el at showed that melanosome cargo can be transferred to fibroblasts to stimulate tumor niche formation (Dror et al., 2016). Melanosomes were selectively enriched for a particular set of miRNAs when the mature and pre-mature vesicles were compared to malignant melanocytes during melanoma initiation, depicting a potential function in paracrine signaling. An additional role for melanosomes in melanoma cell homeostasis has been proposed in response to cytotoxic drug treatment (Chen et al., 2006). Melanosomes capture cisplatin, increasing the drug export, and contributing to the therapy resistance of cancer cells. These studies provide evidence for a link between their normal function in the dermis/epidermis and their role in skin cancer, postulating the possibility for malignant cells to profit from physiological cell to cell communication mechanisms.

## EVs as players in the melanoma-microenvironment interplay

**FIGURE 3:**
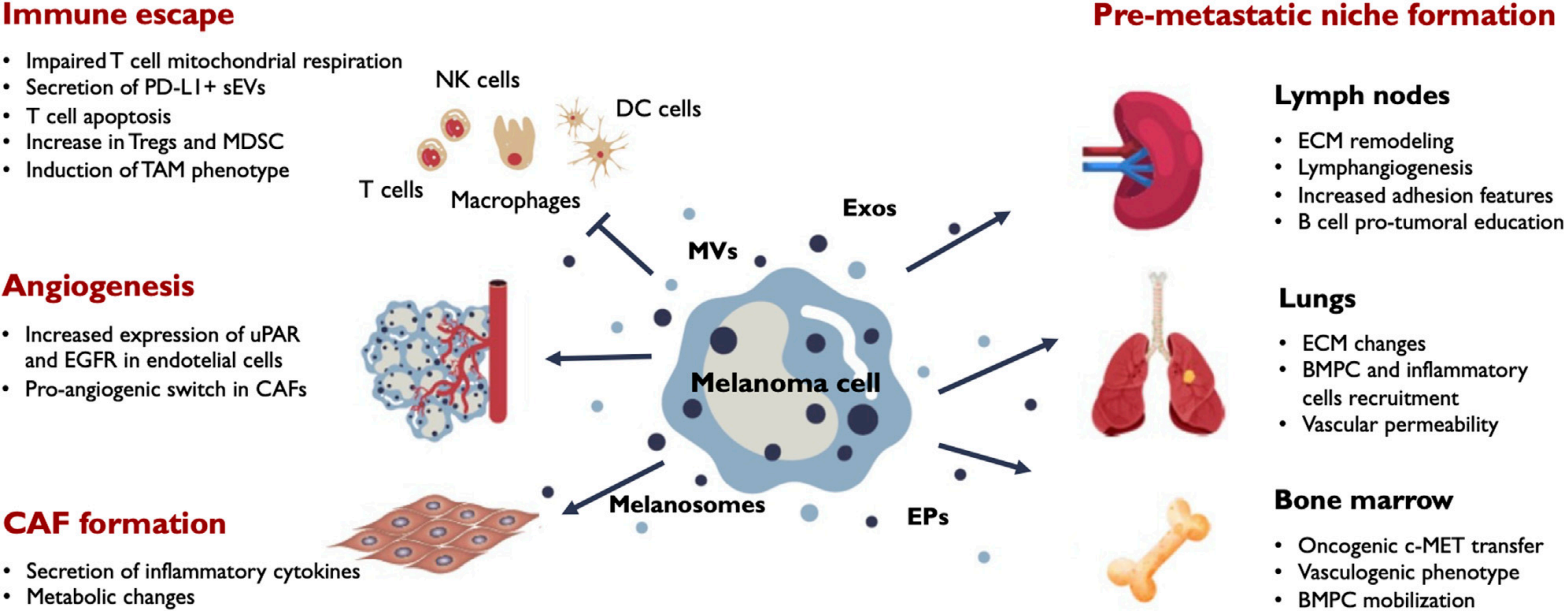
Main outcomes of EV release in melanoma progression. Melanoma cells secrete a variety of EVs including melanosomes, exosomes (Exos), microvesicles (MVs) and extracellular particles (EPs). Secreted vesicles target different stromal and immune populations in the tumor microenvironment and influence their phenotypic behaviour. The main effects induced by melanoma EVs in the tumor milieu include immune tolerance, angiogenesis and CAF formation. EVs also promote phenotypic changes in the tumor draining lymph nodes, the bone marrow and distant organs sucn as the lung contributing to the formation of efficient pre-metastatic niches that facilitate metastatic colonization by melanoma cells. BMPC, bone marrow progenitor cells; CAF, cancer-associated fibroblasts; ECM, extracellular matrix; MDSC, myeloid-derived suppressor cells; TAM, tumor-associated macrophages; Tregs, regulatory T-cells; uPAR, urokinase plasminogen activator surface receptor.

The tumor microenvironment (TME) is the environment around cancer cells. It includes surrounding blood vessels, immune cells, fibroblasts, mesenchymal stromal cells, signaling molecules and the extracellular matrix (ECM). The interactions among these cell types, the structural scaffold and cancer cells are crucial for disease progression and tissue response to the pathological scenario. EVs together with soluble factors play a relevant role in this communication (Figure 3).

The delivery of immunosuppressive or tolerogenic signals to the infiltrating immune cells appears to be a main outcome of EVs in the tumor microenvironment (Whiteside, 2016). For example, TIM-3 shuttled in sEVs impairs CD4^+^ T-cell function and promotes M2 polarization in macrophages (Li et al., 2022b). Melanoma-derived exosomes, enriched for a subset of coding and non-coding RNAs alter the transcriptome of cytotoxic T-cells that impact in mitochondrial respiration (Bland et al., 2018). In addition, exosomes can directly activate the mitochondrial apoptotic pathway of CD4^+^ T-cells through their miRNA cargo (Zhou et al., 2018a), thus affecting two crucial lymphocytes populations. The lack of tumor infiltration by CD8^+^ T-cells is associated with poor patient response to anti-PD-1 therapy. Phosphorylation of hepatocyte growth factor-regulated tyrosine kinase substrate (HRS), a pivotal component of the ESCRT-0 complex, restricts tumor infiltration of cytolytic CD8^+^ T-cells, regulating anti-tumor immunity by inducing PD-L1^+^ immunosuppressive exosomes (Guan et al., 2022). Using mass cytometry, the systemic immune landscape in response to tumor-derived sEVs has been recently characterized. Melanoma-derived sEVs significantly and extensively influenced the composition and intracellular pathways of immune lineage and T-cells, favoring an immunosuppressive environment (Du et al., 2022). Additionally, sEV exposure significantly enhanced the PD-1/PD-L1 axis in CD4^+^ T-cells and myeloid cell subsets.

EV release also contribute to the tumor cell strategies for degrading the extracellular matrix. In fact, release of vesicles carrying metalloproteases such as MMP-2 and MMP-9 by melanoma cells is an important step in the invasive process (Schnaeker et al., 2004). MT1-MMP, another extracellular matrix-degrading enzyme is present in vesicles secreted by melanoma (Hakulinen et al., 2008).

Melanoma-derived EVs can promote cell phenotype transition in the tumor microenvironment. As mentioned above, melanosomes selectively enriched for a particular set of miRNAs including miR-211 are incorporated by stromal fibroblasts. This miRNA targets IGFR2 activating MAPK pathway and contributes to a CAF switch (Dror et al., 2016). Additionally, miR-155 and miR-210 present in melanoma sEVs induced a metabolic reprogramming of fibroblasts increasing aerobic glycolysis and decreasing oxidative phosphorylation (Shu et al., 2018). CAFs are more receptive than their normal counterparts to tumor sEVs, as assessed by increased transcription of genes for inflammation-supporting cytokines and chemokines, namely IL-6 or IL-8 (Strnadová et al., 2022). Mesenchymal stem cells (MSCs) also are influenced by melanoma-derived sEV exposure, suffering an oncogenic reprogramming and PD-1 expression (Gyukity-Sebestyén et al., 2019). Additionally, melanoma cell-derived sEVs modulate bone marrow-derived MSCs (BM-MSCs) phenotype for the production of large amounts of macrophage-recruiting chemokines such as chemokine (C-C motif) ligand 2 (CCL2) and CCL7 (Lin et al., 2016). Furthermore, cancer-derived EVs trigger endothelial to mesenchymal transition of human umbilical vein endothelial cells in an *in vitro* microfluidic model followed by the induction of a CAFs profile (Yeon et al., 2018). All these phenotype changes contribute to a more favorable environment for tumor growth. It has also been shown that CAFs can be reprogrammed towards a pro-angiogenic phenotype through the exosomal transfer of miR-155-5p that activates the SOCS1/JAK2/STAT3 pathway (Zhou et al., 2018b). Another pro-angiogenic sEV-associated molecule is uPAR that has been shown to promote endothelial tube formation *in vitro* and vessel formation using *in vivo* matrigel plug assays (Biagioni et al., 2021).

Tumor-associated macrophages (TAMs) are the most abundant immune cells in the tumor microenvironment, promoting tumor initiation, growth, progression, metastasis, and immune evasion (Simiczyjew et al., 2020). As mentioned above, melanoma-derived sEVs induce a tumor-promoting TAM phenotype in macrophages (Gerloff et al., 2020).

The dynamic interaction between melanoma cells and the stroma affects also to the adipocyte-rich hypodermic layer of the skin. Adipocytes plentifully secrete sEVs, which are then taken up by tumor cells, leading to increased migration and invasion. An increase in the fatty acid oxidation in melanoma cells upon adipocyte sEV exposure might control this pro-metastatic melanoma feature (Lazar et al., 2016). It appears that adipocyte-derived sEVs stimulate mitochondrial metabolism and cause a redistribution of mitochondria to membrane protrusions supporting cell motility (Clement et al., 2020). Drug resistance is usually related with microenvironment interactions, and there are few evidences supporting an EVs role on this process. Small EVs purified from cell cultures derived from cisplatin-treated tumors contained the drug, correlating to the pH conditions of the culture medium (Federici et al., 2014).

## Circulating EVs in plasma, lymph and other fluids

In addition to their local effects within the tumor microenvironment, EVs are able to reach the blood stream and the lymphatic system through unstable endothelial layered vessels and lymphatic capillaries present in the tumor mass. EV circulation through the blood stream and the lymph precedes and accompanies metastatic dissemination and growth.

As it occurs with other tumor-derived EVs, melanoma EV presence in plasma and serum has been broadly demonstrated (Peinado et al., 2012; Mathew et al., 2020; Pietrowska et al., 2021). As mentioned before, a prominent characteristic of plasma-circulating EVs in melanoma patients is their pro-coagulant and immune tolerant properties although platelet-derived EVs and other EV sources could considerably contribute to these features (Laresche et al., 2014; Muhsin-Sharafaldine et al., 2016). For example, the presence and immunosuppression exerted by plasma EV-transported PD-L1 (Chen et al., 2018; Cordonnier et al., 2020) might have extensively been contributed by circulating EVs derived from immune PD-L1^+^ cells.

A more refined method for capturing subpopulations of circulating EVs employs specific antibodies coupled to beads. For example, using immunocapture with anti-CD81 coated beads, not only proteins but also fatty acids have been profiled in plasma-circulating CD81-EVs showing an increase in fatty acid content in melanoma late stages compared to healthy donors (Paolino et al., 2021). Due to the systemic influence of tumor growth through soluble factors and vesicle secretion, tumor-derived vesicles in circulation are just a portion of the total EV content in the blood. There is a growing interest in analyzing tumor-specific EVs in which mutations or other proteins could be selectively increased through tumor progression. One such a method proposed the specific isolation and separation of melanoma-derived EVs from other cell sources (such as endothelium or hematopoietic cells) using anti-CSPG4 affinity-based capture (Sharma et al., 2018). Using that approach, the proteomic comparison of melanoma-derived plasma exosomes with remaining non-melanoma plasma exosomes showed an increase in signaling and immune regulating proteins including LDHA, NOTCH2 and thrombospondin-1 among others. Interestingly, high levels of ALIX and absence or low levels of Contactin-1 in melanoma-specific plasma EVs was associated with disease progression (Pietrowska et al., 2021), although these results should be validated in larger cohorts.

From the clinical point of view, circulating vesicles have risen a lot of expectation as a possible alternative or a complementary approach to other more established liquid biopsy strategies like circulating tumor cells (CTCs) and ctDNA. Although some studies have similar or improved detection of specific mutations in EVs than in ctDNA (García-Silva et al., 2020), there is still much work to do to achieve the robustness and the clinical standards of ctDNA (Yu et al., 2021). On the other side, the heterogenous cargo including DNA and miRNAs present in EVs could also allow multi-marker analysis with increasing sensitivity and specificity *versus* mono-parameter analysis (LeBleu and Kalluri, 2020).

In this context, EV-based surrogate biomarkers of melanoma progression and therapy monitoring have been proposed. Detection of *BRAF* V600E mutation in melanoma patients is improved when analyzed in isolated vesicles compared to the reference protocol for ctDNA isolation (Zocco et al., 2020). *KRAS* mutations have also been reported in RNA from plasma-derived EVs in melanoma patients (Yap et al., 2020). Interestingly, the presence of BRAF splicing variants in plasma due to therapy resistance to BRAF inhibitors could be associated to extracellular vesicles (Clark et al., 2020).

Circulating miRNA are also promising biomarkers and have also shown their association with melanoma progression (Huang et al., 2022). In particular, MV-associated let-7g-5p and miR-497-5p have been suggested as putative predictive markers in response to MAPK inhibitors in melanoma (Svedman et al., 2018). Although mtDNA has also been detected in melanoma vesicles (Cheng et al., 2020) and circulating mtDNA mutations, in particular those in the D-loop have been detected in the plasma of melanoma patients (Takeuchi et al., 2004), this alternative nucleic acid material remains largely unexplored in circulating EVs.

In addition to the blood, EV presence has been spotted in other biological fluids such as the lymph. The interstitial pressure acting in tissues preferentially directs solutes and small molecules to the collecting intra-tumoral lymphatic vessels that display a specific permeable junctional organization (Dieterich et al., 2022). This could provide an explanation for the more elevated levels of EVs in lymphatic drainage than in plasma from melanoma patients (Broggi et al., 2019; García-Silva et al., 2019). Exosomes injected in the footpad of mice have been shown to travel through the lymph and be retained in the popliteal lymph node (LN) (Hood et al., 2011; Srinivasan et al., 2016). Of note, the proteomic profile of sEVs in lymphatic drainage differs from the one obtained from plasma sEVs and constitutes an important contribution to the lymph protein composition. Not surprisingly, in lymphatic drainage-derived sEVs, metastatic and immunomodulatory proteins are abundant (Broggi et al., 2019; García-Silva et al., 2019; Maus et al., 2019). EV-associated nucleic acid cargo appears also to be enriched in lymphatic drainage which could also be used as a prognostic tool in addition to ctDNA. In this regard, presence of BRAF V600E mutation in lymphatic exudate-derived EVs after lymphadenectomy was indicative of fast disease progression (García-Silva et al., 2019), suggesting their use as a highly sensitive method for detection of minimal residual disease in melanoma at early stages. LN metastatic melanoma patients also display significantly elevated levels of metastasis-related miRNA in lymphatic drainage than in plasma indicating that this alternative fluid could reveal a reliable miRNA information about disease progression (Broggi et al., 2019).

The relatively frequent presence of brain metastases in melanoma patients suggests that melanoma-derived EVs could be detected in cerebrospinal fluid (CSF). EV presence in CSF can be due to the ability of EVs to alter and cross the brain-blood barrier (Zhou et al., 2014; Tominaga et al., 2015). In particular, melanoma-derived exosomes are able to induce downregulation of tight junctions in brain microendothelial cells, penetrating the barrier interface and causing glial activation on a brain-blood barrier chip (Wang et al., 2022). The detection of melanoma-associated mRNAs (MAGE-3, MART-1 and tyrosinase) in CSF of stage IV melanoma patients was indicative of subsequent brain metastasis (Hoon et al., 2001). It can be speculated that detected CSF-derived mRNAs could be loaded into EVs. Additionally, CSF is enriched in ctDNA compared to plasma (De Mattos-Arruda et al., 2015) and tumor BRAF mutation has been detected and was informative of disease course in melanoma patients with leptomeningeal involvement (Li et al., 2016; Melms et al., 2018). Thus, it is possible that CSF-derived EVs could be a valuable source of information for melanoma patients with brain metastases or risk to develop them.

In agreement with their heterogeneous content, stability in biofluids and circulation properties, we have provided here some relevant examples of the use of melanoma-derived EVs as biomarkers of melanoma progression. These and other relevant findings regarding EV diagnostic potential has been reviewed recently (Bollard et al., 2020; Kamińska et al., 2021; Lattmann and Levesque, 2022)*.* Finally, the physical characteristics of EVs, together with their modulable specific cargo provide a remarkable source for therapeutic uses such as drug or target shRNA delivery (Olmeda et al., 2021; Hou et al., 2022).

## Melanoma EVs and the lymph node pre-metastatic niche

LNs surrounding primary tumors such as melanoma, breast cancer or prostate cancer are frequently the first places where metastatic spread is detected. Indeed, these tumor-draining LNs are also denominated sentinel LNs (SLNs) as their inspection after lymphadenectomy is indicative of early metastatic dissemination. LN status as determined by histological examination of SLNs for the detection of metastases is the most accurate prognostic factor in melanoma (Faries et al., 2017). Lymphatic dissemination can be considered as an alternative route to blood circulation for metastatic cells at least, for certain tumor types. Accordingly, LN metastasis might be an early step towards distal metastasis since subsequent dissemination from metastatic LNs to distant organs such as the lung has been demonstrated (Brown et al., 2018; Pereira et al., 2018). Furthermore, LN metastatic cells display increased survival compared with tumor cells in subsequent hematogenous dissemination (Ubellacker et al., 2020). This hypothesis is also supported by the fact that the lymph could be a more favorable travel environment than plasma with reduced mechanical and oxidative stress and protection against ferroptosis (Sleeman, 2000; Ubellacker et al., 2020). Recently, it has been suggested that metastatic cells growing in the LNs induce a systemic tolerance mediated by regulatory T-cells that could favor distant metastasis (Reticker-Flynn et al., 2022).

Before metastatic LN colonization occurs, tumor draining lymph nodes (TDLNs) undergo substantial changes that are a consequence of the tumor-LN interplay and that are defined as the LN PMN in homology to the defined concept applied to the cellular and ECM remodeling occurring in distant organs anteceding metastatic growth (Peinado et al., 2017). Key features of the LN PMN are active lymphangiogenesis, fluctuations in the amounts of different immune populations, dilation of high endothelial venules and active proliferation of fibroblastic reticular cells related with alterations in LN architecture and matrix composition (Qian et al., 2006; Sleeman, 2015; du Bois et al., 2021).

TDLNs undergo extensive lymphangiogenesis that generates an expanded lymphatic capillary network (Harrell et al., 2007; Sleeman and Thiele, 2009; Olmeda et al., 2021) and precedes melanoma cell colonization (Harrell et al., 2007; Olmeda et al., 2017). This process is analogous to the one occurring in LNs elaborating immune responses during infection and inflammation. Tumor soluble factors such as VEGF-A and VEGF-C reaching the LNs induce active lymphangiogenesis (Hirakawa et al., 2005; Hirakawa et al., 2007). Cooperating with these soluble factors, tumor small EVs such as exosomes have been shown to be retained in the LNs where they induce LN remodeling at different levels modifying the transcriptional programs of LN cell populations, altering matrix composition and enhancing LN metastatic colonization by tumor cells (Hood et al., 2011; Pucci et al., 2016; García-Silva et al., 2021; Leary et al., 2022) (Figure 3). Lymphatic endothelial cells (LECs) are the population that incorporates more melanoma-secreted sEVs, at least, at early time points of exposure, probably due to their structural distribution within the LN that establish them as the first recipients to the lymph cargo. LECs undergo notable transcriptional and functional changes in response to sEV exposure that includes activation of the lymphangiogenic program (García-Silva et al., 2021; Leary et al., 2022). Those EV-induced lymphagiogenic signals are mediated by nerve growth factor receptor (NGFR) through NF-KB and MAPK pathways and involve the phosphorylation of VEFGR3, a key driver of the lymphangiogenic program. Induction of LN lymphangiogenesis by tumor EVs has also been observed in other cancer types such as colorectal cancer, hepatocarcinoma or cervical squamous cell carcinoma (Li et al., 2018b; Sun et al., 2019; Zhou et al., 2019).

In addition to the activation of the lymphangiogenic program, adhesion molecules such as ICAM-1 and VCAM-1 are upregulated in LECs exposed to melanoma sEVs facilitating subsequent attachment of circulating tumor cells (García-Silva et al., 2021; Leary et al., 2022). Interestingly, sEVs also pervade the peripheric tolerance functions of LECs. EV antigen cargo is cross-presented on MHC-I complexes by LECs inducing apoptosis in CD8^+^ T-cells (Leary et al., 2022).

Subcapsular sinus (SCS) and medullary macrophages also take up sEVs (Pucci et al., 2016; Broggi et al., 2019; García-Silva et al., 2021), as expected by their phagocytic capacity. SCS macrophages appear to act as a barrier for sEV dissemination. Disruption of this barrier during tumor progression allows sEVs to interact with B cells which will confer additional immunity to the growing tumor (Pucci et al., 2016). However, it is unclear if EVs are able to modulate macrophage functions or if their accumulation is responsible for the SCS barrier disruption observed at later times. In this regard, murine melanoma-derived exosomes induced a mixed M1/M2 phenotype with increased Arginase and iNOS expression in RAW 264.7 macrophages (Bardi et al., 2018), suggesting that, similar effects could result in an immunosuppressive macrophage behavior in TDLNs.

Exposure to melanoma-derived sEVs also induces changes in the composition of the LN matrix such as increased levels of laminin 15 and collagen 18 (Hood et al., 2011) and thus, sEVs could be at least partially responsible for the elevated collagen and hyaluronic acid deposition found in the pre-metastatic TDLNs compared to naïve LNs (Rohner et al., 2015). Additionally, active proliferation of fibroblastic reticular cells has also been observed in TDLNs and constitutes another characteristic of the LN PMN (Riedel et al., 2016). Consequently, the conduit network expands altering its size exclusion properties and affecting antigen delivery. It is plausible that tumor-secreted EVs could reprogram stromal populations such as fibroblastic reticular cells, similarly to the influence that they exert on stromal fibroblasts in the tumor microenvironment.

The important structural remodeling that occurs in the LN PMN is accompanied by alterations in LN immune populations associated with immunosuppressive features such as increased DC dysfunction (van den Hout et al., 2017; Cochran et al., 2001), impairment of T-cell capacities (Mansfield et al., 2011) and augmented secretion of immunosuppressive cytokines (Lee et al., 2005; Torisu-Itakura et al., 2007). In melanoma, the effects of EVs on LN immune populations have been barely explored *in vivo*. Interestingly, B16-F10-derived small EVs induced increased numbers of regulatory T-cells in LNs when injected in mice (Du et al., 2022).

In summary, melanoma-derived small EVs are involved in the main changes occurring in the pre-metastatic LNs that surround the primary tumor and together with tumor soluble factors such as VEGF-C, VEGF-A, GM-CSF favor LN remodeling and establish a more suitable environment for metastatic colonization (Figure 3). Remarkably, there are growing evidences that tumor-immune system interactions in the LN setting appear to influence distant metastasis outcome and EVs are remarkable candidates to be involved in that interplay.

## Melanoma EVs and distant pre-metastatic niches

EV physical properties and stability in biological fluids allow for long distance transfer of tumor molecular cargo. These characteristics noticeably fit with the PMN concept in distant organs that establish tumor-derived factors as key elements for pre-conditioning distant organs by altering their cellular homeostasis and ECM structure before tumor cell colonization occurs in order to facilitate or fuel metastatic seeding (Peinado et al., 2017).

Another related concept is tumor organotropism that expresses the selective capacity of cancer cells to target specific secondary organs depending on the primary tumor type (Minn et al., 2005; Hu et al., 2009; Gao et al., 2019). Small EVs play a key role in defining the organotropism of tumors in such a way that sEVs from tumors that metastasize in the lung can partially redirect cancer cells with a different tropism to this organ (Hoshino et al., 2015). The configuration of integrins exposed on the sEV surface determines the selective binding to ECM and cells located in different organs (Hoshino et al., 2015; Becker et al., 2016). For example, α6β4 and α6β1 integrins predispose to lung colonization and αvβ5 favors liver metastasis. Skin melanoma does not display a clear organotropism but shows multi-organ metastatic capacity including metastasis to lung, liver, skin and brain. Remarkably, the integrin profile in EVs secreted by several melanoma cell lines indicates a broad expression of the integrin protein family (García-Silva et al., 2021) that could suggest a non-specific tropism pattern or, in other words, a broad metastatic target range.

In early stages of melanoma, tumor-shed EVs could elicit anti-tumor responses as shown in a poorly metastatic mouse model (Plebanek et al., 2017). In this setup, released exosomes stimulated the expansion of Ly6C^low^ monocytes, the recruitment of natural killer (NK) cells to tumors and eventually, limited lung metastatic colonization. However, in more advanced stages, increased numbers of secreted exosomes and other EVs exert a wide range of immunosuppressive functions through the expansion of Treg and myeloid-derived suppressor cells subsets, impairment of antigen-presentation and cytotoxic T-cell and NK cell dysfunctions (Whiteside et al., 2021). The numbers of NK cells and CD8^+^ T-cells are diminished in the spleen and bone marrow of mice exposed to melanoma-derived sEVs (Du et al., 2022). This agrees with the presence of PD-L1 on the surface of melanoma-secreted exosomes that interacting with CD8^+^ T-cells can inhibit T-cell function (Chen et al., 2018).

Those effects notably decrease the effectivity of adaptive immune responses and fit with the described presence of pro-inflammatory and immune-modulatory proteins and RNAs in the cargo of metastatic melanoma-secreted sEVs (Whiteside, 2016; Bardi et al., 2019). In addition to these *in vivo* studies, there are abundant *in vitro* evidences that support a role for tumor-secreted EVs in the modulation of the immune landscape to create a permissive environment for metastatic colonization. For example, SK-MEL-28-derived EVs promoted a decrease in the DC maturation markers CD86 and CD83 and an impaired secretion of chemokines such as Flt3L, IL15 or MIP-1 (Maus et al., 2017). These effects were mediated by S100A8 and S100A9 proteins present in the EV cargo.

In healthy lungs, melanoma-derived EVs downregulate IFNAR1 and its downstream target cholesterol 25-hydroxylase which prevents EV uptake. This IFNAR1 downregulation facilitates EV message amplification by an increase vesicle uptake (Ortiz et al., 2019). Neutrophils are also recruited to the lung PMNs by the action of the RNA cargo in melanoma-derived sEVs. Lung epithelial cells activate TLR3 in response to exosomal RNAs that promotes the secretion of chemokines attracting neutrophils to those areas (Liu et al., 2016).

In addition to these local and systemic effects of EVs, exosomes can influence bone marrow progenitor cells (BMPCs) through the horizontal transfer of c-MET and induce their differentiation towards a vasculogenic phenotype (Peinado et al., 2012). Exosomes also promote BMPCs mobilization to the lungs of melanoma-bearing mice by increasing pro-inflammatory molecules such as S100A8 and S100A9 and ECM remodeling and thus contributing to the establishment of efficient PMNs (Figure 3). Small EVs can also act locally in the distant organ microenvironment by increasing vascular permeability that could favor the tissue entry of inflammatory cells (Becker et al., 2016).

Although melanoma cells can disseminate to organs other than lung, few studies have addressed the requirements and mechanisms for the formation of suitable PMNs in those locations. It is possible that sEVs from different tumor types but with similar tropism opt for parallel strategies. For example, brain-tropic breast cancer cells secrete exosomes carrying CEMIP protein that targets brain endothelial and microglial cells inducing endothelial cell branching and inflammation in the perivascular niche. In this way, breast cancer sEVs favor vascular co-option for tumor cells (Rodrigues et al., 2019). In melanoma, a pro-inflammatory phenotype is induced in astrocytes exposed to melanoma exosomes that included the upregulation of IL-1α, IL-1β, CXCL1 and CCL2 among others (Gener Lahav et al., 2019). In the liver PMN, pancreatic adenocarcinoma-derived sEVs loaded with migration inhibitory factor (MIF) target Kupfer cells (Costa-Silva et al., 2015). sEV-modulated Kupfer cells induce the secretion of TGF-β by hepatic stellate cells promoting and inflammatory and fibrotic environment. Interestingly, *in vitro* studies with different melanoma cell lines suggest that osteotropic melanoma-derived sEVs induce CXCR7 expression in non-osteotropic cells and promote the SDF-1/CXCR4/CXCR7 axis (Mannavola et al., 2019). This strategy could be advantageous for the establishment of a pro-tumor PMN in the bone.

## Future directions

In this review, we have focused on melanoma-derived EVs providing an analysis of most relevant information on the characterization of their cargo and roles in tumor progression. Due to space constrains, the review does not include an exhaustive discussion about EVs from stromal and immune populations in the microenvironment that would act in coordination with melanoma EVs for fueling tumor progression. Particular attention can be dedicated to EVs released by activated platelets such as MVs or microparticles that are able to promote angiogenesis and metastasis of several lung cancer cells (Janowska-Wieczorek et al., 2005). EVs derived from CAF populations also influence tumor cells and favor their metastatic behavior. However, the complex network of effects between tumor, immune and stromal-derived sEVs is far from being understood.

EVs have been shown to play crucial roles in different aspects of cancer cell migration, like directional sensing, cell adhesion, ECM degradation, and leader-follower behavior (Luga et al., 2012; Sung et al., 2015; Sung et al., 2021), but the mechanisms are not fully understood. Their implications for melanoma local and distal metastasis deserve more attention.

Tumors induce other systemic outcomes that are observed in late/terminal stages of the disease such as thrombosis, myelopoiesis or cachexia. It is not unreasonable to speculate that those devastating consequences of the terminal stages of cancer could also be at least in part mediated by tumor-derived EVs and experimental evidences are beginning to be provided in several tumor types (Yang et al., 2019; Raimondi et al., 2020; Miao et al., 2021).

Another relevant question relates to EV heterogeneity that theoretically would allow a vast complexity of paracrine and long-distance cell-to-cell interactions. However, it is plausible that depending on the cell type and the physiological/pathological status, only certain types of vesicles are released or predominate among the EV pool. The EV content might also be influenced by therapeutics (Lunavat et al., 2017; Svedman et al., 2018). Undoubtedly, more efforts would be required to thoroughly understand this biological exchange system in the cancer context.

In conclusion, we are on the road of understanding the communication mechanisms between tumor cells and the patient’s body. Melanoma is a cancer with a remarkably enhanced EV release that has extensively contributed to unravel the role of EV-mediated tumor-environment cross-talk. Indeed, EV release exerts multiple functions in benefit of melanoma progression. A profound knowledge of these mechanisms could lead to the development of novel strategies to block melanoma progression.
