# Supplementary material for: Extracellular vesicles and melanoma: New perspectives on tumor microenvironment and metastasis
Source: Front Cell Dev Biol. 2023 Jan 10;10:1061982. doi: 10.3389/fcell.2022.1061982 (PMC9871288; doi:10.3389/fcell.2022.1061982)
Supplement: Supplementary file 1 [file DataSheet1.PDF]

Table 1. Cargo of melanoma-derived EVs

Cargo	Findings
**mRNAs**	
TOP1	Enriched in EVs derived from human melanoma cells (A375 and SK-MEL-28) compared to primary melanocytes (61)
ABCB5, TYRP1	Reduced in EVs derived from human melanoma cells (A375 and SK-MEL-28) compared to primary melanocytes (61)
CXCL1, CXCL2, CXCL8	Enriched in EVs from human melanoma cells (A-375, SKMEL-28, and C-32) compared to primary melanocytes (65)
PD-L1	Reduced in plasma EVs in patients responding to anti-PD-1 treatment, and increased levels in subjects with disease progression (66)
**Proteins**	
p120 catenin, Nedd5, PGRL, ezrin, radixin	Identified in exosomes derived from human melanoma cell lines (SK-MEL-28 and MeWo) (69)
Annexin A1, annexin A2, syntenin-1, HAPLN1	Enriched in EVs from the human metastatic melanoma cells A375 compared to primary melanocytes (61)
CD44, MAPK4K, GTP-binding proteins, ADAM10	Enriched in the human metastatic melanoma cell line FEMX-I (71)
FAK1, SRC, Rac1, RhoA, Cdc42, EGFR, EPHB2	Enriched in exosomes derived from human metastatic melanoma cells (A375M and 1205Lu) compared to non-tumorigenic (MNT-1, G-1 and 501mel) and tumorigenic but non-metastatic cells (Daju and SKMel-28) (70)
TYRP2, VLA-4, HSP70	Enriched in exosomes from plasma of melanoma patients with advanced disease compared to healthy subjects (17)
MIA, S100B	Enriched in exosomes isolated from the sera of stage IV melanoma patients compared to healthy controls (78)
CD14, PON1, PON3, APOA5	Different content in EVs from plasma of melanoma patients at different stages (0-IV) (79)
PD-L1	Its level on EVs correlates with therapeutic response to immune checkpoint inhibitors in melanoma (82)
PD-1	Its enrichment on melanoma EVs was suggested as a possible resistance mechanism (81, 83, 84)
MCSP, MCAM, LNGFR, ErbB3	EV level changes after targeted therapies in melanoma patients (85)
**miRNAs**	
let-7c, miR-138, miR-125b, miR130a, miR-34a, miR-196a, miR-199b-3p, miR-25, miR-27a, miR-200b, miR-23b, miR-146a, miR-613, miR-205, miR-149, and others	Enriched in human melanoma cells (A375) compared to melanocytes (61)

miR-214-3p, miR-199a-3p, miR-155-5p	Enriched in exosomes of human melanoma cell lines (A375, MML-1 and SKMEL-28) (87)
miR-100-5p, miR-99b-5p, miR-221-3p, miR-24-3p, miR-125b-5p, and others	Enriched in human melanoma cells (WM9, WM35 and WM902B) compared to melanocytes (88)
miR-222	Delivery of miR-222 by EVs from metastatic melanoma cells increases the tumorigenicity of less aggressive melanoma cells (94)
miR-106b-5p	The transfer of this miRNA by exosomes to melanocytes promotes *in vitro* cell migration, invasion, and *in vivo* lung metastasis (97)
miR-21	Exosomal miR-21 from murine B16 melanoma cells delivered to stromal fibroblasts increases MMP expression, favoring melanoma progression (98)
miR-92b-3p, miR-182-5p, miR-183-5p	Enriched in melanoma versus melanocyte derived EVs and in the plasma of melanoma patients compared to healthy donors (88)
miR-17, miR-19a, miR-21, miR-126, miR-149	Enriched in plasma EVs of patients with metastatic sporadic melanoma compared with unaffected control subjects (92)
miR-125b	Reduced levels in exosomes obtained from serum of patients with advanced melanoma (100)
miR-532-5p, miR-106	Enriched in exosomes from serum of melanoma patients compared to healthy individuals (101)
miR-191, let-7a	Enriched in EVs from stage-I melanoma patients compared to healthy individuals (93)
**lncRNAs**	
Gm26809	The transfer of this exosomal lncRNA from melanoma cells to fibroblasts reprograms fibroblasts into cancer-associated fibroblasts (103)
**DNA**	
$BRAF^{V600E}$	Exosomal mutant BRAF as a promising biomarker for monitoring BRAFi therapy (104); Detection in lymphatic drainage derived EVs from stage III melanoma patients correlates with risk of relapse (77)
**Lipids**	More saturated and shorter fatty acid chains in EVs from low metastatic cells (LCP) compared to high metastatic (SKMel28) melanoma cells (106)
**Metabolites**	Decreased levels of palmitoyl carnitine, sphingosine 1-phosphate, elaidic carnitine, phosphatidylcholines, phosphatidylethanolamines, and glycosphingolipid ganglioside GM3 in exosomes from serum of melanoma patients compared to that of healthy individuals (108)
